# ‘Early Introduction’ of Cow’s Milk for Children with IgE-Mediated Cow’s Milk Protein Allergy: A Review of Current and Emerging Approaches for CMPA Management

**DOI:** 10.3390/nu15061397

**Published:** 2023-03-14

**Authors:** Caoimhe Cronin, Yukta Ramesh, Carlo De Pieri, Roberto Velasco, Juan Trujillo

**Affiliations:** 1 Department of Paediatrics and Child Health, University College Cork, T12 DC4A Cork, Ireland; 2 Department of Pediatrics, Azienda ULSS2 Marca Trevigiana, 31100 Treviso, Italy; 3 Gerencia de Atención Primaria Valladolid Este, 54, 47010 Valladolid, Spain; 4 HRB Clinical Research Facility Cork (CRF-C), Irish Centre for Maternal and Child Health Research (INFANT), Cork University Hospital, T12 DC4A Cork, Ireland

**Keywords:** cow’s milk protein allergy, children, tolerance, immunotherapy

## Abstract

IgE-mediated cow’s milk protein allergy (CMPA) is one of the most prevalent food allergies in early childhood. Though the cornerstone of management involves the strict avoidance of milk products while awaiting natural tolerance, research increasingly shows that the rates of resolution are slowing down. Therefore, there is a need to explore alternative pathways to promote tolerance to cow’s milk in pediatric populations. This review aims to combine and appraise the scientific literature regarding the three CMPA management methods: avoidance, the milk ladder, and oral immunotherapy (OIT) and their outcomes in terms of efficacy, safety, and immunological effects. Cow’s milk (CM) avoidance virtually protects against allergic reaction until natural tolerance occurs, with hypoallergenic substitutes available in the market, but accidental ingestion represents the main issue for this strategy. Introduction to baked milk using the milk ladder was designed, with most CMPA patients successfully completing the ladder. Similar to baked milk treatment, many OIT protocols also demonstrated decreased IgE and increased IgG4 levels post protocol, as well as a reduction in wheal size diameter. Though these strategies are shown to be safe and effective in CMPA, future clinical trials should compare the safety and effectiveness of these three management strategies.

## 1. Introduction

Food allergy is emerging as a significant public health concern, affecting individuals of all ages, ethnicities, and socio-economic strata [1,2]. The prevalence of food allergies varies slightly by region. In the United States, greater than 10% of the population likely suffers from at least one IgE-mediated food allergy, and in Ireland, 4% of infants are affected by allergies—similar to other European countries [1,2]. Moreover, the prevalence of food allergy has been increasing in recent years [3]. Cow’s milk protein allergy (CMPA) is one of the more common food allergies, with a prevalence of 1% in children in Ireland. Internationally, studies using self-reporting to determine the prevalence of CMPA found the prevalence ranged from 1.2–17% [4].

CMPA usually presents before 6 months of age and may resolve spontaneously in childhood [5]. However, research increasingly shows that the rates of resolution are slowing down, meaning that the allergy can persist into adulthood [6]. The rates of CMPA in breastfed children have been shown to be lower than in formula-fed children, at 0.5% [5]. Possible risk factors for developing cow’s milk protein allergy include premature birth, birth by caesarean section, maternal food allergy, antibiotic during pregnancy, and the introduction of complementary foods when the child is less than 4 months of age [7,8].

The diagnosis of CMPA is mainly based on the patient’s history and physical examination. Tests that may be conducted include Skin Prick Tests (SPT) and serum-specific IgE. These tests have low specificity but high sensitivity. Hence, they may be positive even in non-allergic individuals. In the event that CMPA is suspected, cow’s milk should be eliminated from the diet for 1 month, followed by the reintroduction of cow’s milk to the diet. Children with IgE-mediated CMPA should be re-evaluated every 6–12 months to check for tolerance to cow’s milk protein [5]. Although an oral food challenge (OFC) has been deemed the gold standard for CMPA diagnosis, its practicality in diagnosing CMPA for children has been debated. Some systematic reviews and international guidelines have suggested the use of cut-off SPT wheal sizes and specific IgE values to diagnose cow’s milk allergy, without the need to conduct OFCs [9]. For instance, the British Society of Allergy and Clinical immunology (BSACI) suggested that infants under 2 years of age with wheal size ≥ 6 mm during cow’s milk SPT are 100% specific for a positive challenge, and OFC is not recommended [10].

Allergies can be IgE or non-IgE mediated. Symptoms of the allergic reaction typically present within 2 h of ingesting the allergen when they are IgE mediated [11]. Non-IgE-mediated symptoms are slow onset, taking between hours to days to present [5]. For the purpose of this review, when CMPA is mentioned, it refers to the IgE-mediated allergy. The symptoms can be cutaneous (70–75%), gastrointestinal (13–30%), respiratory (1–8%), or cardiovascular in nature. They can range from urticaria (hives), diarrhea, vomiting, angioedema, and wheezing to anaphylaxis (1–4%) [5].

There are 30–35 g of protein per liter of cow’s milk (CM), with at least 20 proteins being potential allergens. Whey proteins (soluble proteins) make up 20% of CM, and caseins (insoluble proteins) make up 80% of CM. Most CMPA is caused by whey proteins, but the allergy can be further exacerbated by caseins. The major milk allergens are casein (alpha-s1-, alpha-s2-, beta-, and kappa-casein) and whey proteins—β-lactoglobulin (Bos d 5), as well as α-lactalbumin (Bos d 4). Minor allergens, which have been shown to cause allergy in only a small number of patients, include immunoglobulin, bovine serum albumin, and lactoferrin. CMPA patients are usually sensitized to more than one allergen, with sensitivity to both whey and casein proteins [5,12].

Living with food allergies can have significant psychosocial impacts on the patient. The occurrence of post-traumatic stress symptoms is also greater in those who have experienced anaphylaxis in the past [3]. A study on 10–16-year-olds revealed that those with food allergies have an increased likelihood of facing separation anxiety, anorexia nervosa, depression, and generalized anxiety [13]. Moreover, as children transition into adolescence and college life and begin to exercise their independence, parents are less able to monitor their food habits. A survey conducted on college students showed that merely 40% followed their dietary plan, and only 6.6% carried their adrenaline auto-injector with them [3]. Dairy products are nutrient dense and are recommended as part of healthy eating guidelines and in particular as a source of both protein and bone-supporting nutrients such as calcium in young children. Implementing a milk-avoidance diet could also cause nutritional deficiencies if not managed appropriately. Additionally, the management of food allergy can also be a financial burden [14].

Three methods have been extensively trialed for the management of CMPA: milk avoidance, home introduction of milk using a stepwise strategy called “the milk ladder”, and oral immunotherapy (OIT). This review aims to combine and appraise literature regarding the three CMPA management methods and their outcomes in terms of efficacy, safety, and immunological effects. The literature search and preparation of this review were conducted according to the guidelines on the writing of narrative styles of review outlined by Ferrari [15].

## 2. Avoidance of Cow’s Milk

Natural tolerance has been attributed to the cytokine profile of T cells and its influence on B cell class switching—from prominent IgE production to IgG4 production—increasing tolerance [16]. Alpha and beta caseins have a configuration of IgE-binding epitopes that are more three-dimensional (3D) in young children than in adolescents and adults. Incomplete maturation of the digestive system in children could leave 3D configuration relatively intact, exposing the infant to sensitization [17].

Avoidance of CMP is achieved by using alternative milk formula products, including extensively hydrolyzed formulas (eHF) and amino acid-based formulas (AAF). eHF and AAF work by reducing the immunogenicity of the CMP epitopes, changing the 3D structure of the antigens present in CM.

### 2.1. The Acquisition of Natural Tolerance

**Figure 1:**
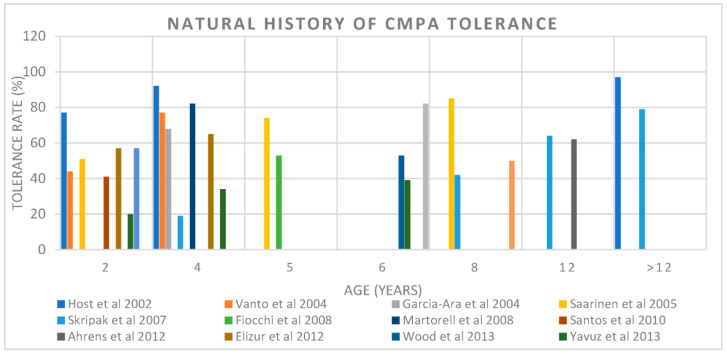
Natural History of CMPA tolerance according to age across studies [18,21,22,23,24,25,26,27,28,29,30,31].

The natural history of most CMA patients is characterized by spontaneous acquisition of tolerance before 3 years of age [16]. It has been found that 56% of children formerly diagnosed as CMA became tolerant at one year of age, and 77% at two years of age [18,19]. Moreover, less than 0.5% of adults are reported to be allergic to CM [20]. Avoidance of the trigger food is considered the first and obvious strategy to implement in CMPA, awaiting natural tolerance. Traditionally, it was thought to be a transient allergy with a high rate of resolution in childhood. However, the resolution rate is not heterogeneous across studies (Table 1 and Figure 1). The increasing persistence of CMPA must be taken into consideration when discussing management and treatment.

Dietary avoidance limits the symptoms of CMPA, and it is also part of the diagnostic work up of all patients with suspected CMPA. For patients who experienced life-threatening events, this approach would be considered the “common sense” approach, but because the clinical picture related to CMPA can vary, most patients present with vague symptoms and unclear clinical history, requiring a trial of CM avoidance to confirm the diagnosis.

### 2.2. Evidence of the Effectiveness of CM Avoidance

Evidence supporting solely CM avoidance is sparse. A systematic analysis performed by the European Academy of Allergy and Clinical Immunology (EAACI) on acute and long-term management of food allergy found only one non-randomized comparison in which eliminating cow’s milk in patients with a positive radioallergosorbent test (RAST) was associated with the remission of symptoms and reduced reactions to allergens over time [35,36]. However, it should be noted that this study evaluated children who were sensitive to cow’s milk and not children specifically with an IgE-mediated CMP allergy. Though dietary avoidance reduces or even eliminates the symptoms, dietary restrictions should keep in consideration the patient’s nutritional needs, ideally with the guidance of a dietician.

### 2.3. CM Avoidance: Evidence from Alternative Formula Studies

Three studies exploring substitutes for CM assess the acquired tolerance to CM [37,38,39]. de Boissieu and Dupont found that after consuming AAF for a mean duration of 11.4 ± 7.9 months (range, 3.5–41), 41 of 52 children (78.8%) had achieved tolerance to CM by the end of the observational period, with the median age of CMP tolerance being 20.5 months [37].

The two most recent studies cited here explore the role of the gut microbiome in the development of tolerance to CM [38,39]. Canani et al. compared the occurrence of allergic manifestations and subsequent acquisition of tolerance to CM in children with a median age of 5 months consuming either eHCF with the probiotic Lactobacillus rhamnosus GG (LGG) or eHCF alone. Though the incidence of tolerance increased in both groups, the incidence was significantly higher in the eHCF + LGG group than in the eHCF alone group (*p* < 0.01 at 12 months) [38]. This study provides evidence that changes in the developing gut microbiome in early life may speed up the acquisition of tolerance to CM. In the most recent study, Chatchatee. et al. observed the development of tolerance of children aged <13 months receiving AAF including synbiotics or AAF alone [39]. Overall, 49% and 62% of subjects were CM tolerant by 12 and 24 months, of treatment respectively. However, no significant difference was found between the two groups. The rates of tolerance were found to be in line with the reported tolerance development trajectory [39]. Though the outcomes of these studies may be conflicting, the role of the gut microbiome in the development of tolerance to CMPA should not be underestimated.

It is clear that determining the resolution of CMPA through dietary avoidance is difficult because of the heterogeneous study populations and different methodologies and the several factors affecting the resolution of CMPA, including age, severity of initial reactions, and presence of multiple food allergies or other comorbid atopic conditions [39]. It is still important to discuss natural CMPA resolution in order to compare the effectiveness of new CMPA management strategies. The outcomes of the above studies exploring milk avoidance interventions are summarized in Table 2.

## 3. Home Introduction of Milk Using a Stepwise Strategy Milk Ladder

### 3.1. The Milk Ladder and Baked Milk

**Figure 2:**
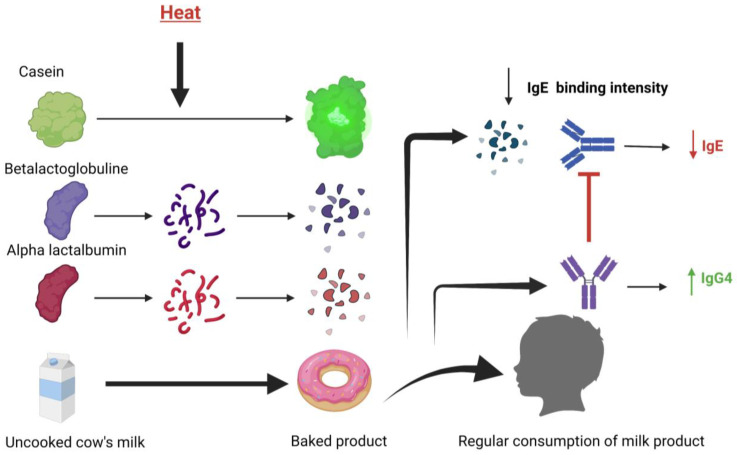
The mechanism of the baked milk ladder. Foods on the milk ladder undergo varying degrees of heat during the baking process. The major milk allergens, casein, betalactoglobuline, and alpha-lactalbumin, are denatured to varying degrees, with casein being the most heat resistant. This baking process alters the stability of these allergens and renders them less allergenic through decreased IgE binding. Regular consumption of these baked milk products causes the increased production of IgG4, which blocks the IgE binding to the allergen. Regular consumption of these baked milk products therefore decreases IgE levels and increases IgG4 levels, allowing for increased tolerance of milk proteins as the degree of allergenicity increases through the milk ladder.

The baking process alters the structure of different milk allergens, changing their stability and subsequently creating decreased allergenicity (through decreased IgE binding) [12]. In many cases, this is because of the destruction of conformational epitopes (antigenic determinants that bind with the IgE receptor) of milk proteins. Children with transient milk allergy are considered likely candidates to tolerate baked milk products [40]. Several studies have reported a good tolerance response to baked milk (BM) introduction in children, even reporting to accelerate tolerance to fresh milk [41,42]. This is supported by the observation that an increase in the intensity of IgG4 binding to CM epitopes occurred concurrently with a decrease in IgE-binding intensity among patients who recovered early from CMPA [43]. The production of IgG4 induces tolerance by blocking the binding of specific IgE to allergen [43]. Therefore, it can be hypothesized that the gradual introduction of denatured epitopes of baked milk proteins promotes the production of IgG4, thus inducing tolerance in milk-allergic patients (Figure 2).

### 3.2. Exploring the Introduction to Milk Using Baked Milk

One of the first studies in the literature to explore baked milk tolerance as a predictor of milk tolerance was by Nowak-Wegrzyn et al. in 2008 [41]. This study challenged the current standard of strict milk avoidance as the primary treatment for milk allergy, as the authors showed that patients who were tolerant to baked milk in an oral food challenge (OFC) showed significantly smaller immunologic reactions to fresh milk after 3 months of ingesting baked milk [41].

A further four studies explored the introduction of baked milk as a treatment for milk allergy from 2011–2018 [40,42,43,44,45]. The amount of patients who became tolerant to unheated milk at the end of the studies ranged from 54% to 88.1% mean (62.5%) [42,45]. This compares to the range of those who underwent strict avoidance for the duration of this trial, which ranged from 0% to 66.7% (mean 21.42%) of those who tolerated unheated milk [42,45]. For these figures, the populations that were chosen to introduce baked milk to their diet had passed a baked milk OFC, whereas the groups who were instructed to strictly avoid milk were those who had failed a baked milk OFC. A strength of the study by Nowak-Wegrzyn A. et al. (2018) was that the authors gave a further unheated milk OFC to those who had passed the baked milk OFC, as those who passed this OFC were instructed to introduce all forms of milk and dairy into their diet, and those who did not were instructed to introduce a baked milk diet. It was not established by the other studies whether those who tolerated baked milk may also tolerate unheated milk during an OFC.

The three studies assessing home introduction to baked milk concluded that treatment of milk allergy using the introduction of baked milk was overall a safe method in patients [40,44,45]. It was found by Dunlop et al. that 35% of patients experienced an adverse reaction during treatment, the majority of which (77%) were characterized as mild [45], and Nowak-Wegrzyn et al. (2018) stated that no adverse reactions to baked milk at home were treated with epinephrine [44].

The literature on the home introduction of baked milk as a treatment for CMPA is limited, but it establishes that this approach is safe and effective in patients who tolerate baked milk. Each study gave detailed instructions to participants to begin home introduction with a baked milk product, a muffin in most cases, in increasing doses, and once tolerated, less baked goods should continue to be introduced in a sequential manner, from a pancake to baked cheese and, finally, to fresh pasteurized milk. However, instruction on the introduction of baked milk varies greatly, with up dosing periods ranging from every 2 months [45] to every 12 months [44]. These studies paved the way for the creation of patient-orientated guidelines for the stepwise progression from extensively heated to less heated milk in the home, known as the milk ladder.

### 3.3. The Creation of the Milk Ladder

The first published milk ladder was the Milk Allergy Primary (MAP) guideline in 2013 [46], indicated for mild to moderate non-IgE-mediated cow’s milk allergy. This is a 12-step approach focusing on common British foods. An international (iMAP) version was published in 2017 in response to the increased uptake of the MAP Milk Ladder internationally [47]. This updated version takes healthy eating, feeding practices across the world, and other food allergies into account [47]. In doing so, they reduced the number of steps and removed high-sugar foods as necessary steps [47].

These ladders were designed with the aim of managing non-IgE-mediated food allergy, and therefore, a complete strategy and guideline using a milk ladder for IgE-mediated CMPA has not yet been established. However, its empirical use in several countries has increased in the last decade. A 2017 publication cited that 60% of physicians previously surveyed acknowledged using the MAP and the IMAP ladder for IgE-mediated allergies [48]. With the introduction of this ladder, other allergy societies, such as the Canada allergy society, have decided to adapt and create their own ladders [49]. This four-step ladder approach was adapted to include foods that were commonly consumed in Canadian households and is intended for use by preschool children with a history of mild IgE-mediated reactions to milk.

### 3.4. Investigating the Effectiveness of the Milk Ladder for IgE-Mediated CMPA

The first study published regarding the use of the milk ladder in IgE-mediated disease was conducted by Ball et al. in the United Kingdom [50]. This was a retrospective study on the use of an adapted milk ladder management for IgE-mediated CMPA. The strategy uses four stages that start from baked milk products (biscuit) with increases to higher amounts with a progression of volume and levels of baked milk through all stages [50]. By stage four, the products are uncooked, and the ladder is finished with the introduction of typical pasteurized milk. Of the 86 patients retrospectively recruited, only 8 patients did not achieve tolerance of all dairy products.

Forty-three percent of patients presented with a mild to moderate allergic reaction during the milk ladder management, with no anaphylaxis diagnosis that required intramuscular adrenaline [50]. These reactions did not inhibit progress through the ladder, as parents reduced the baked milk dose as instructed and continued the progression again. The authors highlighted good compliance by the patients, with very few episodes of accidental or inappropriate diet exposure [50].

Their approach during this milk strategy included the use of not only baked milk products, but the necessity of low doses of each product at the beginning of each stage. This was a process of approximately 5 weeks, with the first introduction to baked milk being that of malted biscuits. The first phase starts with the ingestion of only a small crumb per day for a week (about 0.35 mg of milk protein), and then increasing the amount until a total of between 23 to 43 mg of milk protein is consumed [50]. The authors strongly encouraged starting reintroduction with very low doses and increasing slowly and with caution at each stage, and only progressing to a lesser baked stage once a substantial amount of the product at each stage could be tolerated [50]. They also promote a flexible approach to home-based introduction, eliminating the need for fixed recipes and measured doses that can be tiresome and may hinder progression and allow for varying factors such as the age of the child, lifestyle choices, parental choices, and food availability while maintaining safety [50].

### 3.5. A Prospective Analysis of the Milk Ladder and Strategies of Improvement

The latest study to evaluate the use of the milk ladder as a method of home introduction to milk for those with IgE-mediated CMPA was conducted by d’Art et al. and published in April 2022 [51]. Though this was a randomized trial to evaluate the progression through the milk ladder of infants who received a single dose of the elicited dose of milk (ED05) compared to the control group who did not receive a single dose of ED05, this is also the first published prospective study on the effectiveness of the MAP milk ladder for infants with IgE-mediated CMPA. Unlike previously described trials, this study excluded children who were already tolerating baked milk.

As a group overall, 37/57 (65%) were on step 6 at 6 months, and 13/57 (23%) were on step 12 at 6 months. This improved to 47/57 (82%) on step 6 and 31/57 (54%) at step 12 at 12 months. However, 24/37 (65%) of the intervention group had completed the ladder (step 12) compared to just 7/20 (35%) of the control group by 12 months, showing a significant difference in the rate of progression (chi sq 4.7, *p* = 0.03). The possible reason for this is the administration of the ED05, providing the parents with the confidence to initiate and progress through the ladder. They concluded that the very act of giving infants a single low dose of cow’s milk in the presence of their mothers promoted parental confidence in home introduction, leading to accelerated progression up the milk ladder. This is supported by previous single-dose studies of ED05 of peanut and milk; it was evident that recruited families gained significant support and increased confidence from their participation [52]. This assumption was further supported in the study when it was shown that for the intervention group overall, there was a significant difference in maternal state anxiety between scores at baseline (*M* = 37.5, SD = 12.9) and at 6 months (*M* = 31.5, SD = 8.6); (*t*(32) = −2.81, *p* = 0.008), whereas no significant difference was found in the control group overall for maternal state anxiety for scores at baseline (*M* = 33.1, SD = 8.5) and at 6 months (*M* = 31.7, SD = 11.6); (*t*(14)4.17, *p* = 0.59) [51].

Though it was to be expected that some children would have mild symptoms when transitioning to a higher step on the milk ladder, no serious or unexpected adverse reactions occurred in children progressing through the ladder [51]. Three accidental exposures to milk occurred over the course of the study, all of these happening outside the home in childcare settings and relatives’ houses [51].

Importantly, this is the first study to assess changes in maternal anxiety and food allergy quality of life (FAQOL) and its effect on progression through the milk ladder. This study shows for the first time that maternal trait anxiety was inversely associated with milk ladder progress in both groups, with poorer outcomes in children whose mothers had higher trait anxiety levels. Food Allergy Quality of Life scores improved in all groups by 12 months [51].

**Figure 3:**
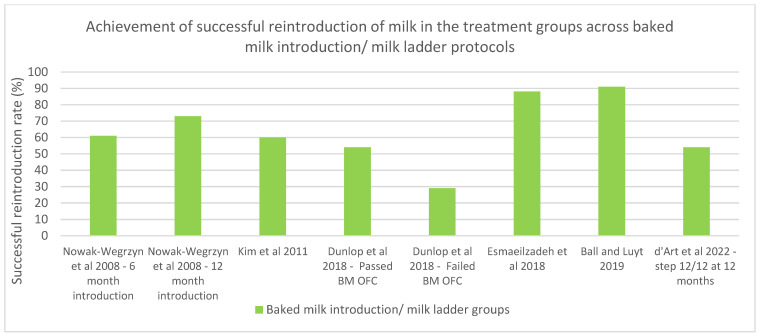
Rates of successful reintroduction to milk using baked milk/milk ladder protocols [40,42,45,46,51,52].

It can be concluded from the abovementioned studies that home introduction to milk using the baked milk ladder is an effective method of inducing tolerance to cow’s milk proteins in the paediatric population, which thus improves food allergy quality of life [40,41,44,45,50,51]. However, with much of the treatment taking place in the home, the need for parent compliance is paramount to the success of the ladder [50]. For successful progression through the ladder to be achieved, a flexible ladder with multiple food choices at each step is needed, with occasional follow-up consultations occurring at key stages in the ladder to allow all families to incorporate the milk ladder into their daily lives [50]. With maternal anxiety correlating with progression through the milk ladder, methods to reduce anxiety, such as an initial dose of fresh milk and starting each step with the smallest amount of milk product, e.g., crumbs, should be encouraged [50,51]. The rates of the successful introduction of milk achieved in this study using the milk ladder protocol and in the previous studies discussed are displayed in Table 3 and graphically displayed in Figure 3.

## 4. Oral Immunotherapy

Standard protocols of OIT consist of different phases. After diagnosis through a positive OFC, the maximum dose of milk that can be consumed is found. Following this, a maintenance phase with daily consumption of the maximum tolerated dosage is conducted for 6–9 months. At the end of the maintenance dose, an OFC is performed testing desensitization. After a variable period of allergen withdrawal, a second OFC is performed testing sustained unresponsiveness. However, recently, it has been proposed that a lower-dosage endpoint is still enough to lower the risk of accidental ingestion with higher compliance and lower adverse events [53].

It is understood that OIT involves a reduction in mast cell and basophil mediator activation. However, the precise immunological mechanism is still to be established. The current evidence available is as follows. Repeated and frequent stimulation of basophils and mast cells results in a desensitization with increased tolerance of milk dosage. This stimulation produces a FoxP3+ Tregs production of cytokines such as interleukin (IL)-10, transforming growth factor (TGF)—β, and interferon (INF) ɣ [54,55,56]. As a consequence, specific IgE levels decrease, and IgG_4_ subclass (anti-inflammatory antibody) levels increase. Initially, B cells produce IgGM+, which then switches to IgG_3_. Next, there is a subclass switch from IgG_3_ to IgG_1_ to IgG_2_ and then finally to IgG_4_ due to alterations in the arrangement of the immunoglobulin-heavy chain locus.

**Figure 4:**
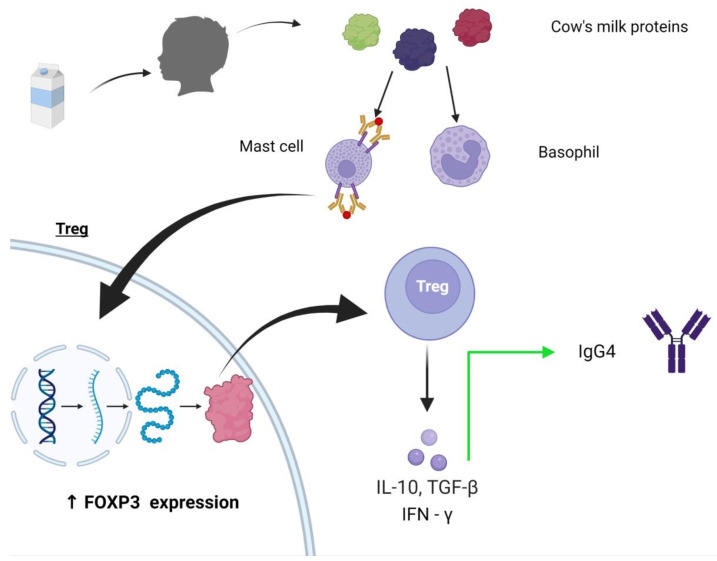
Describing mechanism of oral immunotherapy.

It is believed that repeated allergen exposure, like that which happens in OIT, causes an IgG subclass switch from μ → ɣ3 → ɣ1 → ε producing IgE to μ → ɣ3 → ɣ1 → ɣ2 → ɣ4 producing IgG_4_ [56]. IgG_4_ acts to block antibodies by competing for allergen binding and inhibits the activation of basophils and mast cells. Moreover, the binding of the IgE allergen complex to CD23 is reduced by antigen-presenting cells and acts through FcɣIIb to inhibit IgE levels [57]. Finally, the production of IL-10, IFNɣ, and IL-6 from dendritic cells further depresses the allergic response, leading to tolerance [58]. As a result, T regulatory cell pathways are activated, and T helper 2 cell response is inhibited [59]. The proposed pathway of the achievement of tolerance to cow’s milk proteins is demonstrated in Figure 4.

### 4.1. The Role of OIT and Its Implementation

**Figure 5:**
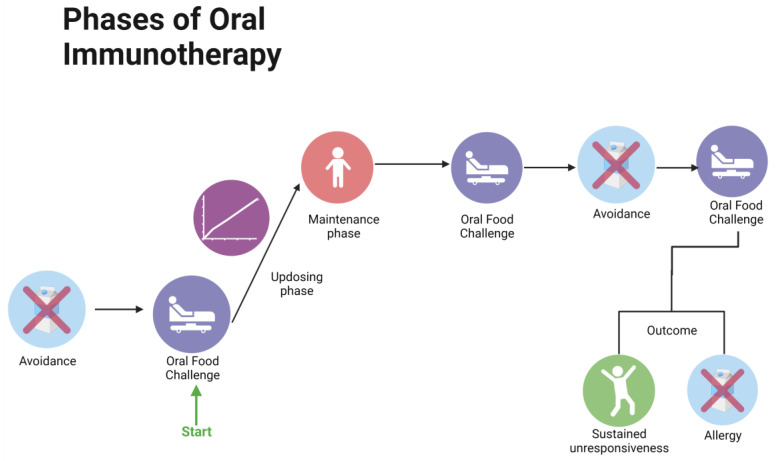
Phases of OIT.

Oral immunotherapy is defined as administering increasing doses of a food allergen (usually in a food vehicle) to an allergic patient in order to increase the threshold at which they react to it [60]. It aims to reduce the risks following accidental ingestion and induce long-term tolerance or sustained unresponsiveness to the allergen. The first reported use of OIT on animals was conducted in 1909 [61]. The general structure of OIT protocols consists of three phases: (i) day escalation, (ii) build up, and (iii) maintenance (Figure 5). During day escalation, six to eight doses of the allergen are administered at short intervals to the patient within the same day, in order to reach a “desensitized state”. This is done under observation in a clinical setting. In the buildup phase, there is daily home administration of the allergen with supervised scheduled dose increases every 1–2 weeks. Once a target dose is achieved, home maintenance commences, and the target dose is consumed daily. The maintenance phase ranges from months to years. An oral food challenge is conducted post protocol to assess for the development of desensitization or sustained unresponsiveness [62].

Currently, the EAACI advises starting OIT for children aged 4–5 years with persistent cow’s milk allergy [63]. Although several clinical trials on cow’s milk OIT have been conducted, a standardized protocol is yet to be established by the EAACI. The trials so far have differed in the type of product, dosage, and duration. All the studies displayed in Table 4 conducted milk OIT trials on children with persistent IgE–cow’s milk allergy. Calvo and Berti et al. conducted OIT on the youngest set of patients, children aged less than 1 year [64,65]. Martorell et al.’s study included children aged 2–3 years, and Efron et al.’s included children aged 1–4 years [66,67]. The remaining studies were conducted on children above age 4. The OIT products used also differed across protocols. Eight studies utilized fresh cow’s milk as the OIT product [64,65,68,69,70,71,72,73]. Takahashi et al. used microwave heated cow’s milk, Mota et al. used pasteurized UHT milk, and Amat et al. compared baked versus fresh milk [74,75,76].

### 4.2. Results of OIT Trials for CMPA Regarding CM Intake

**Figure 6:**
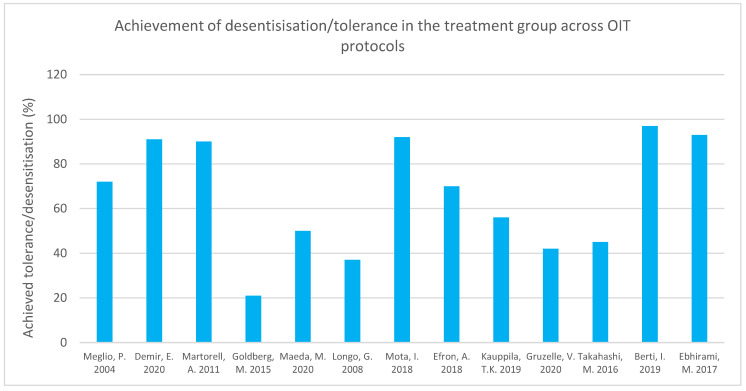
Tolerance/desensitization achievement in treatment groups across protocols [66,67,68,69,71,72,73,74,75,76,78,79,80].

Three studies investigating desensitization/tolerance after OIT all maintained their target OIT dose as 200 mL cow’s milk (CM) [66,73]. Meglio et al.’s study saw 71.4% achieving this dose, and Demir et al., 91.3% [68,73]. Martorell et al. found that 90% of their patients were tolerant to the 200 mL dose, whereas 76.7% of their control group had persistent allergy at the 1-year follow up [66]. Goldberg et al.’s study showed that 21% managed to reach the 1.3 g/day target dose [77]. Maeda et al. found that 50% of their treatment group and none of their control group had a negative OFC 1 year post protocol [71]. Longo et al. noted 37% of their OIT group achieved complete tolerance [70]. Mota et al. reported 92% of patients being able to maintain non-restricted diets following protocol [75]. Efron et al. reported that 70% of the OIT group was able to tolerate all types of milk and dairy products apart from raw milk. In contrast to the others, Efron et al. saw allergy resolution in both the control and treatment groups. However, they found decreased resolution time and age in patients in the treatment group as compared to the control group [78]. In Kauppila et al.’s subjects, 56% could maintain daily milk intake post protocol [67]. Gruzelle et al. noted desensitization of 43% of their patients to baked milk, and Takahashi et al. reported SU in 21% [74,79]. Ninety-seven percent of patients in Berti et al.’s study achieved the target dose of 150 mL [65]. High OIT completion rates were reported by both the oral challenge test group and the fixed starting dose group in a study by Calvo et al. [64]. Ebhirami et al. reported desensitization in 92.9% [80]. Overall, relatively high success rates were reported with OIT, but it is important to note that their target/end OIT doses varied. A summary of the proportion of subjects who achieved desensitization/tolerance to cow’s milk following the OIT protocols in these studies is displayed in Figure 6.

**Table 4:** Studies exploring oral immunotherapy in CMPA.

Author, Country, Sample Size	Population	Intervention	Outcome Reported	Outcome
Meglio P. et al., 2004 [68] Italy 21	Children with severe IgE-mediated cow’s milk allergy, at least 6 years of age	Open-label, six-month protocol. Children administered increasing doses of fresh cow’s milk. Drops of milk diluted in a 1:25 ratio (initial dose = 1 drop). Until day 70, doses doubled every 7 days, and thereafter every 16 days, to achieve a total dose of 200 mL.	IgE measured at 3 months and at end of protocol. End point SPT. Monthly checkups for a minimum of 3 months post OIT. Target dose was achieving 200 mL CM.	A total of 71.4% (15/21) children were desensitized, achieving daily intake of 200 mL CM. A total of 14.3% (3/21) of the children tolerated 40–80 mL/day of undiluted CM, and 3/21 children were unable to follow the protocol due to symptoms arising after CM intake.
Goldberg M. et al., 2015 [77] Israel 15	Patients > 4 years who had failed the milk OIT program were enrolled into the baked milk (BM) OIT	Baseline OFCs were performed, and escalating baked milk muffin doses were administered to determine each patient’s initial OIT dose. A dose below the eliciting dose was administered daily at home if tolerated. Doses were increased by 50% per month under medical guidance. OFCs with unheated milk conducted after 6 and 12 months of OIT.	Target dose was 1.3 g/day baked milk protein. Basophil reactivity and CM-specific IgE measured at 12 months.	A total of 3/14 (21%) were able to reach the target dose of 1.3 g/day baked milk protein. In patients continuing the protocol until 12 months, there was an increase in challenge threshold (*p* = 0.003).
Takahashi M. et al., 2016 [74] Japan 48	Children with persistent CM allergy aged 5–18 years	Initial dose: 1/10th of patient’s threshold dose. Rush OIT phase: microwave-heated CM dose increased by 1.2-fold each time. Ingestion 2–4 times/day at 2 h intervals. Maintenance phase: ingestion of 200 mL daily at home. Rate of SU and desensitization measured 1 year after OIT in both groups. Longer follow up of OIT group. Untreated group on elimination diet post OFC.	OFC conducted after 1 year in the untreated group. Desensitization defined as daily consumption of 200 mL fresh CM without adverse reactions for 2 months. CM-OFC conducted after a week’s milk avoidance period for those achieving desensitization. Blood samples at 1 and 2 years of OIT.	Desensitization achieved in 14/31 (*p* value = 0.002) in OIT group. SU in 21% (7/31) of OIT group at 1 year follow up and by none in untreated group (*p* value = 0.036). Two years post protocol, rate of desensitization and SU (*p* = 0.025 and *p* = 0.008) in OIT group was significantly higher compared to rates one year post protocol.
Skripak et al., 2008 [69] USA 20	Children aged 6–21 with history of milk allergy	Randomization into placebo and OIT group. OIT conducted in 3 phases: (i) build-up day (1st dose = 0.4 mg milk protein, maximum dose = 50 mg); (ii) home dosing with highest tolerated dose and dose increases every 7–14 days; (iii) maintenance dose of 500 mg for 13 weeks. OIT patients tolerating < 2540 mg at last DBPCFC put on avoidance diet.	DBPC, specific IgE, IgG, IgG4, and SPT conducted after OIT.	Post OIT, the median cumulative dose causing a reaction in OIT group was 5140 mg. In the placebo group, all patients had a reaction with 40 mg (*p* value = 0.0003). Median change in milk dose threshold in OIT group post OIT was 5100 mg (*p* value = 0.002). Six of seven patients choosing to undergo open-label OIT after this protocol increased their median threshold dose to 8140 mg from 40 mg (*p* value = 0.03).
Longo G. et al., 2008 [70] Italy 60	Children aged 5–17 with severe allergic reactions and IgE levels > 85 kUA/L	SOTI 2 phase protocol: (i) Rush phase: patients admitted for 10 days and given daily dosing of increasing concentrations of fresh CM until concentration of the solution reached whole milk. Antihistamine given daily. (ii) Home dosing, 1 mL increase every 2nd day until 150 mL of whole milk in single dose reached. Antihistamines used until this point and then tapered off across 4 weeks. Patients asked to continue dairy product intake thereafter.	After 1 year, avoidance group underwent DBPCFC. Specific serum IgE measured at 6 months and 12 months. Measured the number of children tolerating ≥150 mL CM in single dose, children tolerating ≥5 mL in single dose but <150 mL (partial tolerance).	All patients in avoidance group had positive DBPCFC 1 year post protocol. A total of 11/30 in OIT group achieved complete tolerance, many of whom could continue on unrestricted diet (*p* value < 0.001).
Martorell A. et al., 2011 [66] Spain 60	Children 24–36 months old with IgE-CMPA	Diluted doses administered in hospital on day 1 and 2. Thereafter, home administration of fresh cow’s milk twice a day, and doses increased at the research unit once a week for 16 weeks. Diary log kept. No preventative medications used.	Total tolerance defined as ability to consume 200 mL CM, partial tolerance as 20–200 mL CM after 1 year. SPT and IgE measured in both groups at 1 year follow up. Repeat DBPCFC, IgE, and SPT done in those failing desensitization.	In OIT group, 27/30 (90%) achieved 200 mL tolerance and remained tolerant at 1 year follow up. In control group, 23/27 (76.7%) were still allergic at 1 year follow up. Those tolerant continued 200 mL milk intake daily with unrestricted diets. Number needed to treat found to be 1.45.
Amat F. et al., 2017 [76] France 41	Children > 3 years with persistent IgE-CMPA	Patients randomized into (i) baked milk (low-risk) OIT and (ii) raw milk (high-risk) OIT. (i) Doses increased at home every 15 days. Decreased heating of baked milk preparation gradually. When tolerance threshold reached 1970 mg, raw milk used. Daily maintenance dose of 2720 mg. (ii) Increased dosing every 5 weeks in hospital. Highest tolerated dose administered at home daily.	“Responders” defined as tolerating daily intake of 2720 mg milk protein without symptoms at 5 months (for high-risk OIT) or 9 months (for low-risk OIT) follow up. Partial responders as tolerating 340 mg–2720 mg. Serum casein-IgE and IgG measured at end of follow up. Asteir score of the reactions was determined.	At follow up, 104 mg tolerated by sensitive patients, and 1802 mg by others (*p* value = 0.02). After follow up, 36.6% classified as responders, 26.8% partial responders. Average threshold gain was 697 mg. A total of 36.6% remained non-responders.
Maeda M et al., 2020 [71] Japan 28	Patients with CMPA aged 3–12 years	Randomization into OIT and control group. Two-week rush OIT in hospital for OIT group. Gradual daily dose increase. Thereafter, CM intake once a day at home, with dose increase every 14 days by 10–20%. When 100 mL CM reached, this dose was taken daily. Epinastine hydrochloride taken daily. One year of monitoring as outpatients once a month, some with longer follow up. Control group avoided CM for 1 year.	OFC with 100 mL CM and DBCFC conducted after 1 year. SPT, eosinophil count, IgE, IgG4 levels measured. Transcriptome analysis.	Fifty percent of OIT group were desensitized after 1 year. After 1 year, 7/14 in OIT group and 0/14 in control group had negative OFC (*p* value < 0.01). Post protocol, greater CM intake threshold and percentage change in intake threshold in OIT vs. control group (*p* value < 0.01). Seven out of eight followed up 2 years post protocol able to consume >100 mL CM without reactions.
Mota I. et al., 2018 [75] Portugal	Patients aged 2–18 years with persistent moderate-severe IgE CM allergy	Prospective uncontrolled study. Patients who had undergone CM OIT were given 200 mL maintenance dose daily. OIT induction: Doses of unheated and undiluted CM administered, and dose increased at 14–28-day intervals. Minimum follow up of 36 months after maintenance dose reached.	Subjects followed in clinic to check maintenance of 200 mL CM for at least 36 months post reaching the maintenance phase.	Total of 92% of patients able to maintain diet without restrictions and daily ingestion of 200 mL CM.
Efron A. et al., 2018 [67] Israel 110	Children aged 1–4 years passing baked milk challenge started OIT. Control group had patients previously diagnosed with CMPA, and they were followed at different clinics.	Retrospective case-control study comparing children on milk avoidance for 4 years with those treated with extensively heated and baked milk therapy. Home-based 3-month, 4-phase OIT. Daily consumption of a milk product deemed safe at each OFC, e.g., cookie, pizza, etc. Avoidance of milk products other than ones deemed safe. Final OFC: 150 mL raw milk.	OFC with 250 mL unheated CM to determine tolerance.	At follow up, 70% of OIT group able to consume all types of dairy and milk products, but not raw milk. Milk allergy resolution seen in both groups with age. Median resolution age in OIT group = 34 months and in control group = 57 months (*p* value = 0.006). OIT decreased resolution time of CM allergic patients who only had skin symptoms and those with history of anaphylactic reaction to milk. Total of 86% of treatment group achieved tolerance (250 mL of unheated CM) compared to 56% of control group (*p* = 0.003).
Kauppila T.K. et al., 2019 [72] Finland 296	Children ≥ 5 years with IgE-mediated CMPA	Three groups: high dose, low dose, and avoidance. Build-up phase: increasing doses of fresh milk protein administered across 4 months until 6.4 g maintenance dose reached. Antihistamine used in build-up phase. Long-term follow up conducted.	Long-term follow up conducted to measure continuance of daily intake of ≥2 dL CM. Long-term follow-up questionnaire to measure adrenaline use in protocol.	Total of 56% of subjects maintained daily milk dosage of ≥2 dL at follow up (median follow-up duration = 6.5 years).
Demir E. et al., 2020 [73] Turkey 42	Total of 47 patients, 3–13 years old with solely CM IgE allergy selected between 2009–2014	Retrospective cohort study. OFC was considered the initial escalation phase. On 2nd day, a dose 3 doses behind tolerated dose given at home for a week. Build-up phase with fresh CM dose increases in hospital and daily home intake until 200 mL target dose reached (16 weeks). Antihistamines in build-up phase. Dose modified according to reaction.	OFC at 6-month and 1-year intervals to determine tolerance. CM-SPT performed 1 year post OIT. CM s-IgE measured at 6 months, 1, 2, and 3 years of maintenance phase.	Total of 91.3% (42/47) successfully reached target daily dose of 200 mL. Two percent achieved partial desensitization (tolerating 45 mL).
Gruzelle V. et al., 2020 [79] France 63	Children <18 years with CMPA and high casein-specific IgE	Retrospective chart review using baked milk OIT. Initial dose was 1 mL CM. Home increases in dosage until 5 shortbreads reached. This dose taken until 2nd OFC. If positive, the OIT dosing was changed, and another OFC done 1 year later.	Between 1–3 OFCs conducted. Allergic reactions at OFC were graded. sIgE measured.	Desensitization achieved by 42.2% patients in an average duration of 521 days. Increased dose at which patients reacted in last OFC compared to 1st OFC.
Berti I. et al., 2019 [65]	Children < 12 months between 2015–2017 who were admitted to Institute for Maternal and Child Health IRCCS Burlo Garofolo due to hypersensitivity reactions to CM were enrolled in the study.	Initial OFC conducted. Thereafter, OIT was started with home dosing of milk with the highest dose that was tolerated in hospital. Evaluation every 3–4 weeks, and if previous dose was tolerated, dose was doubled and then continued at home. Parents advised to dilute milk into foods commonly consumed by infant. Once higher doses tolerated, dosing increments/frequency were increased. Target dose = 150 mL CM or dairy products with the same amount.	IgE and CM-IgG4 measured at 2 months and then post protocol completion. Target dose of protocol was 150 mL CM/equivalent dairy product. Number of reactions recorded.	Target dose achieved by 66 patients (97%).
Calvo et al., 2020 [64] Spain 335	Children < 1 year with IgE-CM allergy whose parents agreed to OIT underwent this therapy.	Retrospective analysis. Initial oral challenge test conducted on a group of patients between 2007–2011, before starting OIT. From 2011–2018, another set of patients was given OIT without oral food test but with a fixed starting dose of 0.5 mL. OIT: For 1 week, doses given at home twice a day. Infant formula doses mixed with food. Increase in dosage at 7-day intervals in hospital and adapted according to patient. Target dose was 150–200 mL infant formula.	SPT and IgE measured post protocol.	Successful OIT completion in 98.5% of OCT group and 98.1% of FSD group. These patients were able to regularly consume dairy products after this. Median OIT duration in OCT group: 106 days vs. 77 days in FSD group (*p* value = 0.001).
Ebrahimi M. et al., 2017 [80] Iran 14	Children > 3 years old who were supervised at the Allergy and Immunology Clinic Of Children’s Medical Center, Iran for more than 6 months, with a history of CM allergy	DPBCFC conducted. Three-phase fresh milk OIT protocol: rush, build-up, and maintenance phase. (i) Rush phase (1 day): increasing doses of milk administered at 30 min intervals. (ii) Build-up phase: weekly increases in daily milk dose. First dose of the week given as inpatient and the remainder as outpatient. (iii) Maintenance phase (90 days): 200–250 mL CM doses administered per day. Each patient’s doses were given according to severity of their allergy. Patient’s caregivers required to keep a daily log during protocol.	IgE and SPT measured at the end of protocol. Adverse effects recorded following each dose.	Total of 13/14 (92.9%) of subjects completed the protocol and were desensitized to CM.

Three studies described increases in the dose of CM tolerated post protocol in patients undergoing OIT. Skripak et al. noted a significant increase in the milk threshold by 5100 mg in the treatment group after their 3–4-month treatment. Meanwhile, all patients in their placebo group suffered a reaction with 40 mg doses [69]. Maeda et al. found a greater percentage change and CM intake threshold in their treatment group compared to their control group immediately after the 1-year protocol [71]. Similarly, in Gruzelle et al.’s study, patients reacted at higher doses on their last OFC than their first OFC [79].

Apart from desensitization/tolerance achievement, a common and salient result across protocols was adverse reactions. The number of reactions occurring differed in each study. For instance, Gruzelle et al. noted 33.3% of their patients had reactions, and 53% of patients in Goldberg et al.’s protocol were unable to continue OIT due to IgE reactions [77,79]. Mota et al. reported 45% had reactions during their maintenance phase [75]. Skripak et al. noted a significantly higher frequency of reactions among their OIT group (35% per patient) versus their placebo group (1% per patient) (*p* value = 0.02). However, 90% of these reactions required no treatment [69]. In Longo et al.’s study, epinephrine administration was required four times, with two cases needing emergency care [70]. Ebrahimi et al., too, found that two patients needed IM epinephrine administration during OIT treatment, and that rhinoconjunctivitis was the most common reaction following the build-up phase [80]. Calvo et al. concluded in their study that OIT is a safe treatment for CMPA patients, and it should be offered to families immediately after diagnosis [64].

Moreover, some studies found patients who underwent OIT developed long-term tolerance to CM. Longo et al. and Martorell et al., who conducted follow ups 1 year post protocol, found that 11/30 and 23/27, respectively, from their treatment groups remained tolerant to CM and could continue on unrestricted diets [66,70]. Mota et al. reported 92% of patients following unrestricted diets post protocol [75]. Maeda et al. also observed that at the 2-year follow up, seven out of eight patients experienced no reactions when consuming CM doses greater than 100 mL [71,76].

### 4.3. Factors Potentially Influencing OIT Results

Some studies have suggested external factors that potentially affected the results of the OIT protocols. Amat et al. found that patients tolerating a higher CM dose at baseline achieved higher tolerance threshold doses at follow up [76]. These patients achieved a 1802 mg tolerance threshold, whereas more sensitive patients only achieved a tolerance of 104 mg. Mota et al. additionally found a correlation between a patient’s baseline history of anaphylaxis and the allergic reactions they faced in the maintenance phase (*p* value < 0.001) [75]. Relationships between patients’ immunological profiles and outcome of OIT were also observed. Takahashi et al. reported significant differences in initial serum CM-IgE, casein-sIgE, and β-lactoglobulin-sIgE levels and asthma severity between those in the treatment group who achieved sustained unresponsiveness and those that did not [74]. Kauppila et al. found that a patient’s baseline IgE level affected OIT protocol continuance [72]. Five studies additionally administered antihistamines, which may have impacted the number of adverse reactions occurring during the protocol [70,71,72,73]. However, Meglio et al. showed that eight patients achieving target doses remained asymptomatic even 2 months after stopping cetirizine [68]. Interestingly, Maeda et al. conducted transcriptome analysis after specific antigen stimulation on two patients who were non-responders and two patients who were responders to their OIT protocol. Certain milk-specific genes that were up/down regulated were identified in each group. This study has suggested that the identified genes could be chosen as antigen-specific markers to predict OIT outcomes in advance. This is an area requiring further research, as the sample size used by Maeda et al. is too small to be conclusive [71].

Table 4 summarises the outcomes of studies exploring oral immunotherapy interventions for CMPA.

## 5. Early Introduction of Milk Using an OIT Strategy in Young Infants

### 5.1. Early Studies in Early Introduction

As described, the use of CMPA OIT is commonly started in patients between 4–6 years, with little research done in patients less than one year old. The first introduction of this type of management was in 2011 by Reche et al., who performed a case-control study of 20 patients comparing strict avoidance and an OIT protocol in children with a mean age of three, with a one-year follow up [81]. After this period, all OIT patients were tolerant to milk in comparison to three that were tolerant in the control group (*p* = 0.003) [81]. Despite the small sample size, this study created a foundation for larger and more rigorous study of the use of early introduction to milk in young infants in later years.

In the years following, three more studies have investigated the use of early introduction OIT in young infants (Table 5). Lee et al. compared children with challenge-proven CMPA between 7 and 12 months of age who underwent oral desensitization to milk and those who underwent strict avoidance of milk for 6 months [82]. This study again suggests that introducing milk at an early age may induce tolerance in children who may potentially not acquire tolerance naturally as they grow older [82].

### 5.2. Establishing Early Introduction in Young Infants as a Management Strategy for CMPA

The previously mentioned study by Berti et al. explored the efficacy of an early introduction protocol designed to be conducted at home for children aged >12 months. This involved children taking, every day for three to four weeks, the higher dose of milk already tolerated in hospital during an OFC [65]. If parents reported a steady tolerance of the dose of milk offered at home, a doubling dose of milk was administered under medical supervision. If the doubling dose was tolerated in the hospital, parents were instructed to continue offering the same doubled dose at home for another three to four weeks until the next hospital evaluation. Every increase in the dose of milk was initially tested in hospital, in order to favor child safety, until a tolerance of 40 mL of milk was stably achieved by infants. Once the infant had reached tolerance to 40 mL of CM without reactions at home for at least two weeks, families were instructed to increase the dose by 5 mL every week, up to 50 mL tolerated, then to increase the dose by 10 mL every week, up to 100 mL, and then 10 mL every 3 days, up to 150 mL of milk [65].

A total of 66 (97%) of children reached the target of 150 mL milk, with a median time to achieve the protocol of 5.5 months (IQR: 4.5–7, range: 3.5–16) [65]. The study found a high rate of compliance among families and suggests that a home-based introductory protocol for young infants may prove to be a safe and effective solution for families compared to the usual method of strict avoidance [65].

The largest study to evaluate the effectiveness of early introduction using an oral immunotherapy protocol in young infants was conducted by the previously mentioned Calvo et al. [64]. In this retrospective analysis conducted between 2007 and 2018, 335 infants under 1 year of age with IgE-mediated CMPA were treated with early introduction to milk at the moment of diagnosis [64]. Two methods of early introduction were explored. Between January 2007 and June 2011, an in-hospital oral challenge test (OCT) was performed, with gradual increases in infant formula doses, seeking the threshold dose for the start of treatment (OCT group). From July 2011 to June 2018, OIT was introduced with a fixed starting dose (FSD = 0.5 mL), without prior challenging (FSD group) [64]. The starting dose was administered at home at least twice a day for a week. Patients who did not suffer any adverse reactions made dose increments every half week, whereas patients who suffered frequent adverse reactions were proposed to make a 2-week dose increase, with a tolerated dose of 150–200 mL infant formula considered to be a successful treatment [64].

A total of 67 patients in the OCT group (98.5%) and 262 (98.1%) in the FSD group completed the treatment successfully and continued to consume dairy products regularly. The median duration of immunotherapy was 106 days (range 27–269) in the OCT group and 77 days (range 77–254) in the FSD group, showing a significant difference between groups (*p* value 0.001) [64].

Reche et al. found only one infant needed to be pre-treated with antihistamines, and four out ten patients had very mild side effects over the course of the 12-month OIT protocol [81]. During the study by Calvo et al., 45.4% presented some type of adverse reaction, none of which were considered severe, with most of these reactions happening in the hospital during the up-dosing visit [64]. Furthermore, no patient was required to attend the emergency room after home treatment [64].

These findings show that even in the earliest days of infancy, early introduction using oral immunotherapy is an effective management strategy for CMPA and can be safely performed in the home [81].

### 5.3. Early Introduction or the Natural Development of Tolerance?

Finally, the main point of discussion regarding early introduction to milk in young infants is whether the patients were going to achieve a spontaneous resolution of CMPA disease with or without this treatment. The two studies that featured control groups of infants undergoing strict avoidance to milk found a significant number of children achieved tolerance after undergoing early introduction compared to the control at the end of the trial [81,82]. However, it was not established whether the children in the control group would later develop a natural tolerance to cow’s milk. The three studies that have considered this subject concurred that it is too early to know, and that further long-term data and new research are needed to establish the effectiveness of early introduction to milk compared to the development of natural tolerance [64,65,82].

Table 5 summarises the outcomes of studies exploring methods of early introduction of milk using oral immunotherapy protocols for CMPA.

## 6. Discussion

The scientific literature on CMPA has continuously increased since the 1930s [12]. This has led to new treatment strategies that have been a feature of clinical practice in recent decades [12]. A summary of the milestones in the development of treatment for CMPA is shown in Table 6.

To objectively compare the effectiveness of each of the three strategies is not possible at this stage due to the lack of clinical trials comparing these treatments in the management of CMPA. However, the effectiveness of the treatment in resolving CMPA depends on multiple factors.

Dietary avoidance is easy in theory but challenging to be applied. It involves extensive involvement of the dietician to provide guidance on foods to avoid that contain traces of cow’s milk. Even if labeling of food with proper indications is mandatory by law in most countries, including the EU, accidental exposure still occurs. Contamination of food in restaurants, canteens, and other settings is possible. One study followed 80 patients with CMPA until the achievement of tolerance or up to the age of 18 years, finding accidental ingestion of milk in at least a third of them [85]. In addition to the constant risk of anaphylaxis, the avoidance of milk may have significant nutritional implications [4]. Sinai et al. compared adult height in 87 patients with CMPA compared with 36 individuals with no dietary limitations and found that patients with lifelong CMPA had an average 3.8 cm lower adult height than controls [86]. Patients with CMA also have higher rates of vitamin D deficiency [87].

The milk ladder has been shown to be a safe and effective method of introducing baked milk and thus promoting the acquisition of milk tolerance [50]. Though earlier studies suggested that efficacy depends on severity of CMPA [45], with only baked milk-tolerant patients considered for gradual reintroduction, the most recent studies have shown that the milk ladder is safe in children with an allergy to uncooked pasteurized milk [2,50]. Though it can be suggested that the milk ladder improves quality of life as a home-based treatment using readily available milk products, progression through the milk ladder requires a high level of parent compliance and sufficient education and reassurance regarding mild allergic symptoms that may appear as the child progresses through the ladder [50]. Parental anxiety is a significant factor in progression, and a single-dose challenge is a cost-effective and time-efficient manner of reducing this anxiety [2]. Though the safety of the milk ladder as a form of introduction to cow’s milk protein has been shown by several studies, further prospective cohort studies are necessary to determine whether this method merely introduces milk as the child is acquiring natural tolerance, or whether progressing through the milk ladder actively immunomodulates the CMPA in these children who would otherwise continue to be sensitized to cow’s milk.

Oral immunotherapy provides a comprehensive and systematic method of attaining tolerance through four key stages: escalation, up dosing, maintenance, and withdrawal, with high rates of success reported in most studies. A starting dose of milk is established through an oral food challenge, and exact dose regimens are provided for each stage, which may prove easier to follow than the milk ladder for some families, where the quantity of milk proteins given at any time may vary due to differences in cooking preparation and serving provided. However, parents and patients must commit to at least three oral food challenges over the course of the treatment, which may increase the burden of treatment on families compared to the milk ladder, which can be entirely provided for and monitored at home. Early introduction is a novel way of introducing milk to young infants using an OIT protocol [64,65,81]. However, the practicality of this strategy may be a barrier to implementing it in some clinics, where waiting lists to attend allergy specialists may be up to a year long, and waiting periods for oral food challenges even longer in many cases, and many children would not have the opportunity to undergo OIT in infancy.

A significant factor when considering treatments for CMPA is safety and the risk of severe allergic reactions. Though mild allergic symptoms are frequent findings in the milk ladder and OIT protocols, the incidence of severe allergic reactions from accidental exposure to milk is far less than those who are strictly avoiding milk. Parents should, however, receive education from allergy specialists regarding mild allergic symptoms as their child moves to higher doses and should be encouraged to continue progressing through the milk ladder or OIT protocol, and they should be aware of the signs and symptoms of severe allergic reactions [50]. A number of OIT studies reported that a small number of patients required IM adrenaline [70,80], whereas IM adrenaline was not required by any patient due to foods consumed on the milk ladder [2,50]. However, further prospective studies must be carried out in order to investigate whether there is a significant difference in the safety of these two treatment strategies.

## 7. Conclusions

This review of the literature discussed the mechanism, efficacy, and safety of three important management strategies of IgE-mediated CMPA in children: complete avoidance, the milk ladder, and oral immunotherapy. Early introduction using an oral immunotherapy protocol was also discussed as an emerging management strategy. Though the milk ladder and early introduction to milk using oral immunotherapy may provide methods of introduction to milk for CMPA, further studies should prospectively compare the effectiveness and safety of these treatments for IgE-mediated CMPA compared to the natural acquisition of tolerance to milk.

## Figures and Tables

**Table 1:** Natural history of CMA in different populations and settings (adapted and updated from Gianetti et al. [12]).

Author, Year	Number of Subjects	Population/Study Design	Tolerance Rate	Age of Tolerance
Host et al., 2002 [18]	39 (24 IgE mediated)	General prospective birth control	56%	1
			77%	2
			87%	3
			92%	5
			97%	15
Vanto et al., 2004 [21]	162 (95 IgE mediated)	Referral retrospective	44%	2
			69%	3
			77%	4
Garcia-Ara et al., 2004 [22]	66 IgE mediated	Referral retrospective	68%	4
Saarinen et al., 2005 [23]	118 (75 IgE mediated)	General prospective birth cohort	51%	2
			74%	5
			85%	8.6
Skripak et al., 2007 [24]	805 IgE mediated	Referral retrospective	19%	4
			42%	8
			64%	12
			79%	16
Fiocchi et al., 2008 [25]	112 IgE mediated	Referral retrospective	52.7%	5
Martorell et al., 2008 [26]	112 IgE mediated	Referral retrospective	82%	4
Santos et al., 2010 [27]	170 IgE mediated	Referral retrospective	41%	2
Ahrens et al., 2012 [28]	52 IgE mediated	Referral retrospective	61.5%	12
Elizur et al., 2012 [29]	54 IgE mediated	General prospective birth cohort	57.4%	2
			65%	4
Wood F. et al., 2013 [30]	293 IgE mediated	Prospective	53%	5.5
Yavuz et al., 2013 [31]	148 IgE mediated	Prospective	20%	2
			34%	4
			39%	6
Schoemaker et al., 2015 [32]	55	EuroPrevall, European population-based prospective	57%	2
Kim, M. et al., 2020 [33]	189 IgE mediated	Retrospective	50%	8.7
Chong, K. W. et al., 2022 [34]	313 (IgE mediated)	Retrospective	81.8%	6

**Table 2:** Studies exploring methods of cow’s milk avoidance.

Author, Country, Sample Size	Population	Intervention	Outcome Reported	Outcome
de Boissieu D. et al., 2002 [37] France 52	Patients with CMPA allergy who do not respond to eHF, median age 5.3 months	Introduction of AAF. OFC to eHF every 6 months until 2 years of age. When the challenge indicated tolerance of eHF or in children above age 2 years, the challenge was performed with CMP once per year.	Growth (height and weight). OFC to eHF and CMP.	AAF was used for a mean duration of 11.4 ± 7.9 months (range, 3.5–41) and found to be safe in all cases. CMP tolerance occurred at <1 year of age in 8 children, 1 to 2 years in 22, 2 to 3 years in 8, and >3 years in 14. Eleven children did not tolerate CMP by the end of the survey period, the oldest being 5.5 years old. The median age of CMP tolerance was 20.5 months.
Canini, R.B. et al., 2017 [38] Italy 220	Children with IgE-mediated CMA with a median age of 5.0 months (interquartile range, 3.0–8.0 months)	Children were randomly allocated to receive eHCF (*n* = 110) or eHCF containing the probiotic Lactobacillus rhamnosus GG (eHCF + LGG) (*n* = 110) groups and followed for 36 months.	Occurrence of at least 1 allergic manifestation (AM) (eczema, urticaria, asthma, or rhinoconjunctivitis). CM tolerance acquisition using an OFC at 12, 24, and 36 months.	The absolute risk difference for the occurrence of at least 1 AM over 36 months was 20.23 (95% CI, 20.36 to 20.10; *p* < 0.001). The absolute risk difference for the acquisition of cow’s milk tolerance was 0.20 (95% CI, 0.05–0.35; *p* < 0.01) at 12 months, 0.24 (95% CI, 0.08–0.41; *p* < 0.01) at 24 months, and 0.27 (95% CI, 0.11–0.43; *p* < 0.001) at 36 months.
Chatchatee, P. et al., 2022 [39] Germany, Italy, Singapore, Thailand, UK, USA 169	Infants aged <13 months with confirmed IgE-mediated CMA	Subjects were randomized to receive AAF including synbiotics (AAF-S) (*n* = 80) or AAF (*n* = 89) for 12 months.	OFC to CM at 12 and 24 months.	At 12 and 24 months, respectively, 49% and 62% of subjects were CM tolerant (AAF-S 45% and 64%; AAF 52% and 59%), with no statistical difference between the groups.

**Table 3:** Studies exploring the use of baked milk introduction and the milk ladder.

Author, Country, Sample Size	Population	Intervention	Outcome Reported	Outcome
Ball, H. and Luyt, D., 2019 [50] UK 86	Children with IgE-mediated cow’s milk allergy presenting with skin and/or gastrointestinal symptoms and skin prick test < 8 mm	Home-based four-step milk ladder beginning with increasing doses of malt biscuits over 5 weeks. The following 3 steps can last from 4 to 6 months, with three formal clinical reviews occurring at 4–6-month intervals.	Tolerance was determined using a 7-scale scoring system based on the milk ladder (0 being no tolerance, 6 being normal diet).	At the final review, only eight patients of 86 were not tolerating almost all dairy products (≤level 4).
d’Art, Y. et al., 2022. [51] Ireland 60	Infants less than 12 months old with suspected IgE-mediated cow’s milk allergy	Group 1: A single dose of fresh cow’s milk of elicited dose (ED05) (0.1 mL) prior to implementing the milk ladder. Group 2: Routine care. Both groups implemented graded exposure to CM (using the 12-step milk ladder) at home.	Milk ladder position (out of 12) at 6 months and 12 months post randomization.	Step 6 at 6 months: Group 1: 27/37 (73%) to Group 2; 10/20 (50%) (*p* = 0.048). Step 12 at 6 months: Group 1: 11/37 (30%) Group 2: 2/20 (10%) (*p* = 0.049). Step 12 at 12 months: Group 1: 24/37 (65%) Group 2: 7/20 (35%) (chi sq 4.7, *p* = 0.03) Cohort overall at 12 months: 47/57 (82%) on step 6 and 31/57 (54%) at step 12, at 12 months
Dunlop, J. et al., 2018 [45] USA 206	Patients who underwent a baked milk OFC from 2009–2014	Retrospective chart review of those who underwent a BM OFC and follow-up treatment. Those who passed the OFC were given instructions to begin a home-based introduction to BM to their diet or strict avoidance. Group 1: Passed BM OFC—BM introduction. Group 2: Failed BM OFC—BM introduction. Group 3: Failed BM OFC—strict avoidance.	Reported tolerance to baked milk (muffin), lesser baked milk (pancake or waffle), baked cheese, and direct milk at final follow-up.	Group 1: 54% were tolerating direct milk, and 19% were strictly avoiding milk. Group 2: 29% progressed to direct milk, and 38% were avoiding all milk products. Group 3: 10% progressed to direct milk, and 85% were strictly avoiding milk at final follow up.
Esmaeilzadeh, H. et al., 2018 [42] Iran 84	Patients 6 months–3 years old with a history of IgE-mediated milk allergy who passed a BM OFC	Case group: Consumed baked milk in the form of muffin for 6 months and then consumed baked cheese in the form of pizza for another 6 months. Control group: Strict avoidance for 1 year.	After 1 year, both groups underwent unheated milk OFC to evaluate unheated milk tolerance at the end of the study.	Total of 88.1% (37/42) of the patients in the case group and 66.7% (28/42) of those in the control group had developed tolerance to unheated milk (*p*-value: 0.018).
Kim, J. et al., 2011. [40] USA 148	Children aged 0.5–21 years with a diagnosed cow’s milk allergy: based on the initial baked milk oral challenge, subjects were categorized as baked milk reactive or baked milk tolerant.	Baked milk-reactive subjects avoided all forms of milk and were offered a repeat challenge ≥ 6 months from the initial challenge. Baked milk-tolerant subjects incorporated baked milk products daily into their diets and after ≥6 months were offered challenges to baked cheese products.	Baked milk-reactive subjects were offered a repeat baked milk challenge > 6 months from initial challenge. Baked milk-tolerant subjects were offered a challenge to baked cheese products > 6 months from initial challenge. Similarly, after ≥6 months, baked cheese-tolerant children were offered challenges to unheated milk.	Baked milk-tolerant subjects: 39 (60%) tolerated unheated milk, 18 (28%) tolerated baked milk/baked cheese, and 8 (12%) chose to avoid milk strictly. Baked milk-reactive subjects: 2 (9%) tolerated unheated milk, 3 (13%) tolerated baked milk/baked cheese, and the majority (78%) avoided milk strictly. Subjects who incorporated dietary baked milk were 16 times more likely than the comparison group to become unheated milk tolerant (*p* < 0.001).
Nowak-Wegrzyn, A. et al., 2008 [41] USA 91	Individuals between the ages 0.5 and 21 years with diagnosed cow’s milk allergy	Baked milk-tolerant, unheated milk-reactive subjects ingested heated milk products for 3 months and were then re-evaluated. Unheated milk-tolerant subjects were instructed to add milk into the diet. Baked milk-reactive subjects were instructed strictly to avoid all forms of milk.	Follow-up SPT and serum-specific IgE and IgG4 to milk, casein, and b-lactoglobulin measured at baseline and at 3 months in baked milk-tolerant, unheated milk-reactive group.	Milk SPT: 8 mm (2.5–19) at baseline and 7 (2–10.5) at three months (*p* = 0.001). Casein IgG4 (mgA/L): 0.54 (0–8.1) at baseline and 1.02 (0.05–14.7) at 3 months (*p* = 0.005).
Nowak-Wegrzyn, A. et al., 2018. [44] USA 170	Children 4 to 10 years of age with diagnosis of cow’s milk allergy	Group 1: Those reactive to baked milk followed strict milk avoidance. Group 2: Those non-reactive to baked milk tried more allergenic (less heat-denatured) forms of milk (MAFM) food challenges with up dosing every 6 months or every 12 months. Group 3: Those non-reactive to non-baked milk tried unlimited milk and dairy in the diet. Group 4: Comparison with strict avoidance.	Challenges were repeated at 6- or 12-month intervals over 36 months.	Group 1: 20% developed tolerance to baked milk, 0% tolerated non-baked milk. Group 2: 61% children in the 6-month and 73% in the 12-month escalation groups tolerated MAFM. Overall, 41 (48%) children who ingested a baked milk diet became tolerant to non-baked milk; no difference was seen between 6 vs. 12 months’ escalations.

**Table 5:** Studies assessing early introduction of milk using oral immunotherapy protocols in young infants.

Author, Country, Sample Size	Population	Intervention	Outcome Reported	Outcome
Reche et al., 2011 [81] Spain 20	Patients with mean age of 3 months diagnosed with IgE-mediated CMPA	Case group: a protocol of oral induction tolerance. Control group: strict avoidance of cow’s milk.	Tolerance to cow’s milk and specific IgE evaluated 1 year after diagnosis.	Case group: All children tolerant to milk at 1 year. Control group: 3/10 were tolerant to milk after 1 year (*p* = 0.003). OTI protocol was completed in a mean of 5.3 months.
Lee et al., 2013 [82] Korea 31	Infants 7 to 12 months old with challenge-proved IgE-mediated CMPA	Case group: oral desensitization. Control group: strict avoidance.	Oral food challenge, specific IgE, and IgG4 at 6 months.	Case group: 14/16 could accept daily doses of 200 mL of CM. Control group: All but 3 dropout patients receiving the elimination diet still showed allergic symptoms at the follow-up food challenge.
Berti et al., 2019 [65] Italy 68	Children < 12 months of age with a clinical history of IgE-mediated CMPA and SPT and IgE suggestive of CMPA	Home immunotherapy protocol	Number of children were able to take a dose of 150 mL of CM without reactions.	Sixty-six infants (97%) reached the target of the protocol. The target of the protocol was achieved in a median time of 5.5 months (IQR: 4.5–7, range: 3.5–16).
Calvo et al., 2020 [64] Spain 335	Infants under 1 year of age diagnosed with CMA-IgE	Initial dose was administered at home at least twice a day for a week. An in-hospital dose increase was scheduled every 7 days.	Number of children who reached a dose of 150–200 mL of infant formula.	Total of 67 patients in the OCT group (98.5%) and 262 (98.1%) in the FSD group completed the treatment. Median duration of immunotherapy was 106 days (range 27–269) in the OCT group and 77 days (range 77–254) in the FSD group, (*p* value 0.001).

**Table 6:** Milestones of the development of treatment of CMPA.

1909	First recorded use of OIT in animals.	[61]
2001	Allergenic IgE and IgG antibodies for b- and k-casein and alpha(s1)-casein epitopes were identified, with higher levels of these IgE antibodies associated with persistent CMPA.	[17,78,83,84]
2002	A significant number of infants who consume AAF achieve tolerance by 20.5 months.	[37]
2004	Children aged 6 years or more with severe CMPA found to be tolerating a daily intake of cow’s milk following 6-month OIT protocol.	[68]
2008	Significantly smaller SPT mean wheal diameters and significantly greater casein-IgG4 concentrations were shown in CMPA patients who ingested baked milk products for 3 months.	[41]
2011	Subjects who incorporated dietary baked milk were more likely to become unheated milk tolerant.	[40]
	Infants with mean age of 3 months with CMPA who underwent an OIT protocol became tolerant to milk.	[81]
2013	Publication of Milk Allergy Primary (MAP) guideline in 2013 for non-IgE-mediated cow’s milk allergy.	[46]
2017	International version of milk ladder (iMAP) published.	[47]
	Consumption of Lactobacillus rhamnosus probiotic with eHCF promotes tolerance to cow’s milk.	[38]
2018	Clinical trial evidence showing improved tolerance to fresh milk following baked milk introduction compared to strict avoidance.	[42,44,45]
2019	First study to assess the effectiveness of the milk ladder in IgE-mediated CMPA, with most children completing the ladder and tolerating almost all dairy products.	[50]
2020	Large trial in infants under 1 year showing increased tolerance to cow’s milk following OIT protocol.	[64]
2022	Parental anxiety correlates with progression through the milk ladder.	[2]

## Data Availability

No new data was created.
